# Multi-modal fMRI and TMS follow-up study of motor cortical stroke caused by hyaluronic acid filler: A case report

**DOI:** 10.3389/fneur.2022.903648

**Published:** 2022-09-08

**Authors:** Xinwei Tang, Qiurong Yu, Miao Guo, Fan Liu, Yongquan Pan, Jingyuan Zhou, Yue Zou, Cheng Wu, Kewei Yu, Mingxia Fan, Limin Sun

**Affiliations:** ^1^Department of Rehabilitation Medicine, Huashan Hospital, Fudan University, Shanghai, China; ^2^Shanghai Key Laboratory of Magnetic Resonance, School of Physics and Electronic Science, East China Normal University, Shanghai, China

**Keywords:** hyaluronic acid filler, cortical stroke, rehabilitation, fMRI, TMS

## Abstract

**Background:**

Blindness and stroke resulting from hyaluronic acid (HA) fillers are not frequently reported complications. Reports on stroke recovery after HA injection are limited. In the current study, the recovery process, task-based functional magnetic resonance imaging (fMRI), diffusion tensor imaging (DTI), and neurophysiological changes of a patient with monocular blindness and ipsilateral motor cortical stroke after forehead injection of HA are explored.

**Case-report:**

The study comprised a 34-year-old female patient who presented with left eye blindness and a stroke after receiving an HA injection a month before admission. The lesion was mainly limited to the left precentral gyrus, and the patient had pure arm monoparesis. For 3 weeks, the patient received conventional rehabilitation treatments and ten sessions of repetitive transcranial magnetic stimulation (rTMS) intervention. Clinical assessments, neurophysiological evaluation, task-based fMRI, and DTI examinations were conducted to assess her motor improvement and the possible neuro mechanism.

**Clinical rehabilitation impact:**

The patient's right upper limb motor function was almost completely restored after receiving rehabilitation therapy. However, the vision in her left eye did not show significant improvement. The neurophysiological evaluation showed partial recovery of the ipsilesional motor evoked potentials (MEPs). DTI results showed that the ipsilesional corticospinal tract (CST) was intact. Task-based fMRI results indicated that the activation pattern of the affected hand movement was gradually restored to normal.

**Conclusion:**

A case of good motor recovery after stroke due to HA injection with a lesion mainly restricted to the precentral gyrus but without CST damage is presented in the current study. Further studies should be conducted to explore the efficacy and the mechanisms of rehabilitation and neuromodulation approaches to motor cortical stroke.

## Introduction

Medical cosmetology application has significantly increased recently, resulting in several reports of severe vascular complications caused by the facial injection of soft tissue fillers such as hyaluronic acid (HA) and autologous fat. Vision loss and cerebral infarction are rare but severe complications of these injections and can lead to disability and significant effects on the patient's daily life (1). It is challenging to assess the incidence of these complications due to very few case reports and case series. The proposed mechanism of vision loss and cerebral infarction after HA filler injection into the glabella and forehead is the induction of embolism of the terminal blood vessels of the ophthalmic artery and middle cerebral artery by the intra-arterial embolus of filler either anterogradely or retrogradely (2). Previous studies report that facial vascular compromise and neurologic symptoms related to stroke after HA filler injection can be fully or partially abrogated. However, the prognosis of complete vision loss due to an ophthalmic artery or central retinal artery occlusion is often poor (3, 4). Several studies have been published in the journals of ophthalmology and neurology, and most of them mainly report the description and treatment of HA injection complications. Few studies focus on the rebabilitation of patients with stroke caused by HA injection. In this study, the recovery process, task-based functional magnetic resonance imaging (fMRI), diffusion tensor imaging (DTI), and neurophysiological changes of a patient with monocular blindness and ipsilateral hemisphere stroke after forehead injection of HA are reported. The lesion was mainly limited to the left precentral gyrus. The patient had pure arm monoparesis, an uncommon stroke presentation with a reported frequency of <1% of all ischemic strokes (5, 6). Currently, very few studies have explored motor function remodeling and the therapeutic effect of repetitive transcranial magnetic stimulation (rTMS) on motor recovery of pure cortical stroke patients. The present case study sought to explore the possible motor remodeling pattern and recovery mechanism of the patient with precentral gyrus stroke.

## Case description

### Patient

**Figure 1:**
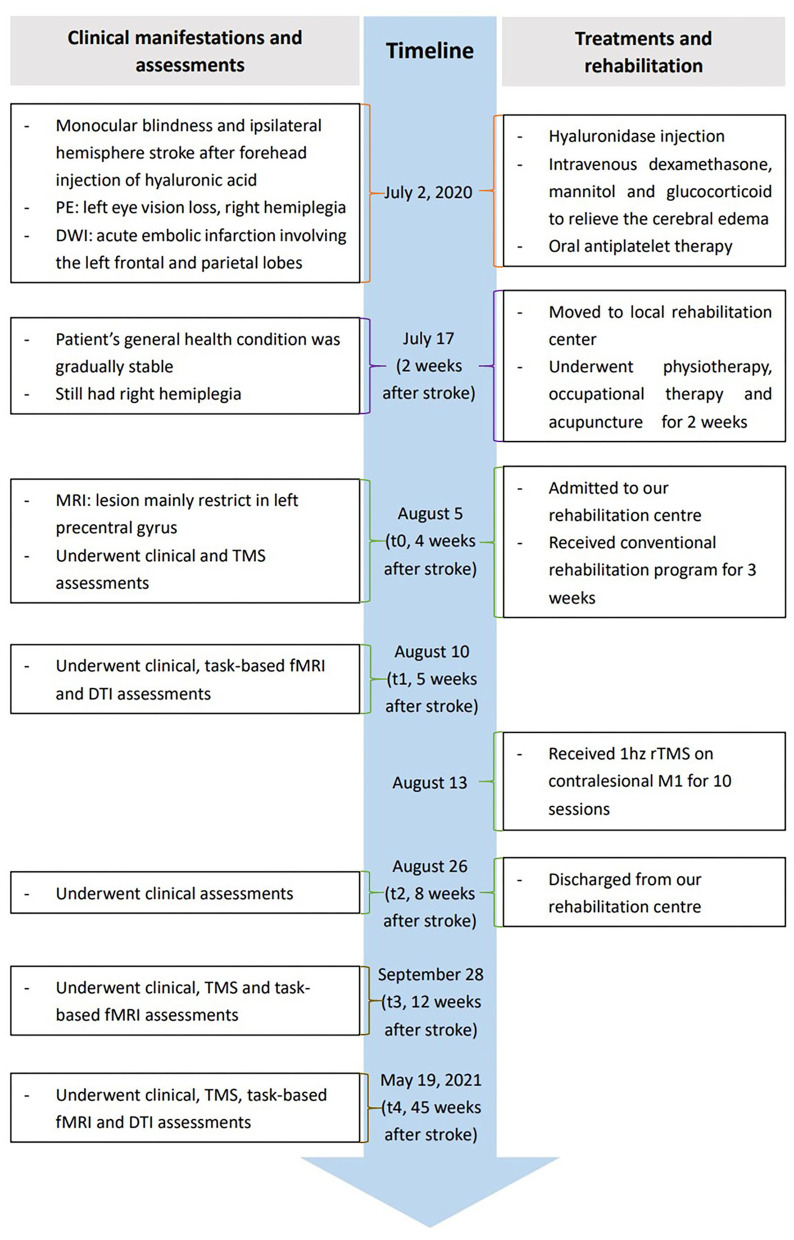
Timeline of relevant data, interventions, and outcomes.

The patient included in the present study was a 34-year-old Asian woman without any medical history related to the present case. The patient reported receiving a 1 mL HA filler injection into her glabella and forehead using a hollow-bore needle at a local private beauty salon. The procedure was conducted by a nurse practitioner without the presence of a physician on July 2, 2020. The patient complained of left periocular pain and complete left eye vision loss shortly after receiving the HA injection. She immediately received a hyaluronidase injection into the left glabella and forehead. The patient presented with nausea, vomiting, headache, and lost consciousness within 10 min. She was taken to a local hospital 1 h post-injection and was admitted to the intensive care unit. She gradually developed muscle weakness in her right limbs. Diffusion-weighted imaging was performed 3 h after the HA injection, which revealed an acute embolic infarction involving the left frontal and parietal lobes. The patient was stable after 2 weeks of conservative medical treatment. The patient was moved to a local rehabilitation center as she presented with right hemiplegia, where she underwent physiotherapy, occupational therapy, and acupuncture for 2 weeks. Her right lower limb muscle strength was restored to normal, and she could walk independently without abnormal gait. However, the motor function in her right upper limb was not significantly improved. The stability and flexibility of her right upper limb and hand were poor, making it difficult for her to hold items with her right hand. Consequently, she was admitted to our rehabilitation center on August 5, 2020, for further observation (Timeline Figure 1, full details in Supplementary material).

### Rehabilitation assessments and treatments

The patient was alert and conscious at the time of admission. MRI showed that the lesion was mainly confined to the left precentral gyrus (Supplementary Figure 1). Clinical assessments, neurophysiological measurements, task-based fMRI, and DTI examinations were performed at different time points to design an individualized neuromodulation protocol and track the process of motor recovery and functional remodeling. The first evaluation, including clinical assessments and neurophysiological analysis, was performed on August 5, 2020 (t0), when the patient was admitted to the rehabilitation center about 1 month after receiving the HA injection. The second evaluation, including clinical assessments, task-based fMRI, and a DTI examination, was performed on August 10 (t1). The third evaluation only comprised clinical assessments and was performed on August 26 (t2) after the patient had undergone conventional rehabilitation treatments for 3 weeks and ten sessions of rTMS intervention on discharge. The fourth evaluation comprised clinical assessments, neurophysiological measurements, and task-based fMRI and was performed on September 28 (t3). The final evaluation included clinical assessments, neurophysiological measurements, task-based fMRI, and a DTI examination and was performed on May 19, 2021 (t4).

#### Clinical assessments

Brunnstrom Stages (BS), Barthel Index (BI), upper limb Fugl-Meyer (UPFM) scale, and Action Research Arm Test (ARAT) clinical assessments were conducted at five different time points to monitor rehabilitation effectiveness and outcomes, as mentioned above.

#### Neurophysiological analysis

Neurophysiological analysis was performed using single-pulse TMS. TMS was conducted using the Yiruide CCY-II TMS instrument (Wuhan, China) with a round coil. Surface electromyography (EMG) was performed by attaching a pair of Ag-Ag/Cl electrodes to the first dorsal interosseous (FDI) muscle of the hand of the patient to assess motor evoked potentials (MEPs). The resting motor threshold (RMT) was determined before stimulation. MEPs were recorded using the self-contained MEP recording system in the Yiruide transcranial magnetic stimulator and analyzed with a coupled MEP-analysis software (Wuhan, China). Ten consecutive MEPs in the cortical representation area of FDI muscles in both hemispheres were recorded as described previously (7). The central motor conduction time (CMCT) for FDI was also recorded (see Supplementary material for further details).

#### fMRI and DTI procedures

High-resolution T1-weighted anatomical images, fMRI BOLD images for affected and unaffected passive finger flexion-extension tasks, and DTI data were acquired using a Siemens Prisma fit 3.0 Tesla MRI scanner (Siemens, Erlangen, Germany) at the Shanghai Key Laboratory of Magnetic Resonance, East China Normal University (Shanghai, China). Details are provided in the Supplementary material.

**Figure 2:**
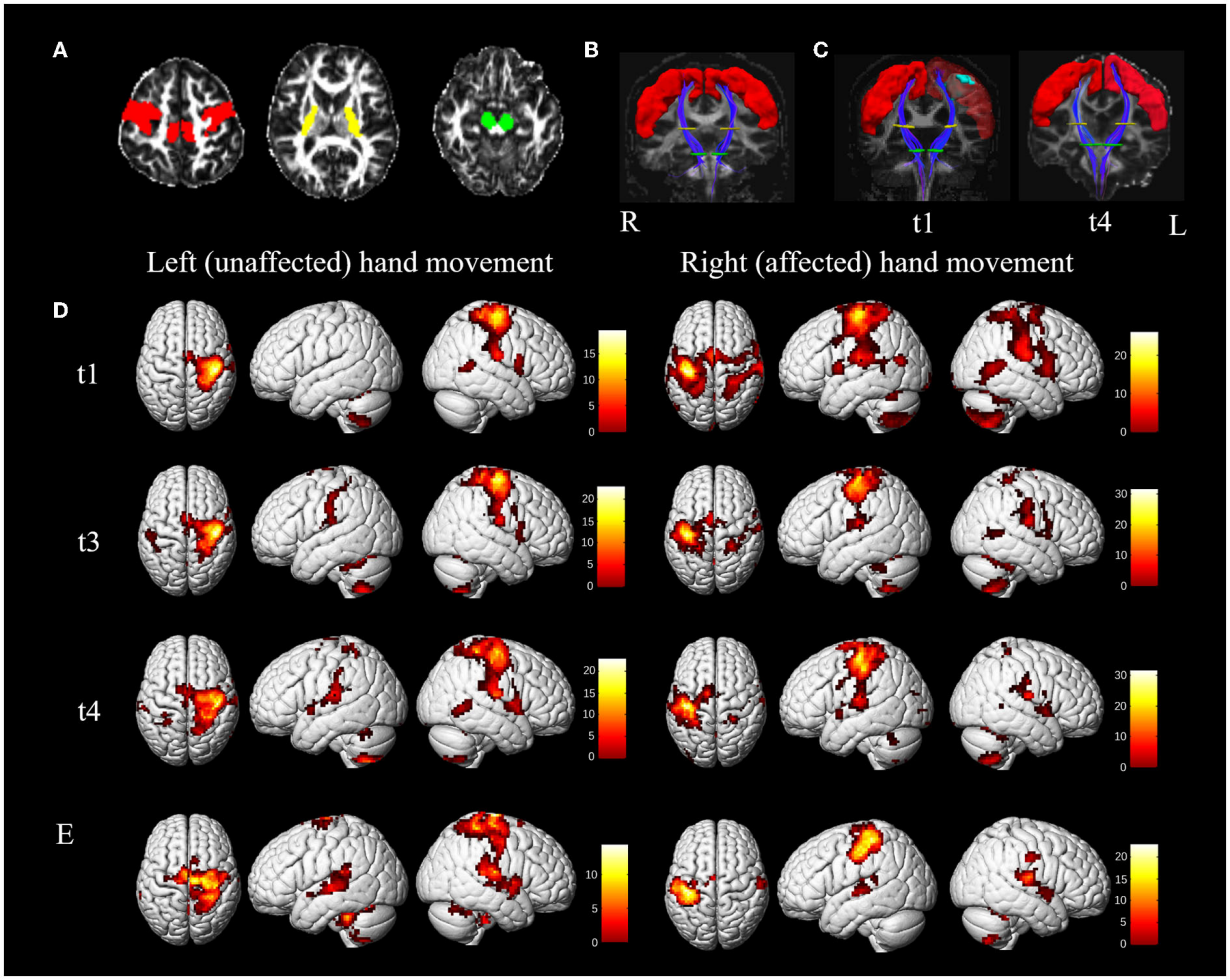
MRI results. **(A–C)**, diffusion tensor tractography of the corticospinal tract (CST). **(A)** Regions of interest (ROI) for reconstructing the CST are located at the precentral gyrus (red color, obtained from the AAL90 template), the posterior limb of the internal capsule (yellow color), and the cerebral peduncle (green color) on the fractional anisotropic (FA) map. **(B)** A coronal view of the bilateral CSTs (blue color) from the healthy control. The red, yellow, and green areas represent the ROI of the precentral gyrus, the posterior limb of the internal capsule, and the cerebral peduncle. **(C)** A coronal view of bilateral CSTs from the patient at t1 (5 weeks) and t4 (45 weeks). On the left of the C map (t1), the cyan region is the lesion registered from T1 space to FA space. **(D,E)** activation during passive unaffected (left) and affected (right) hand movements of the patient and healthy control. **(D)** Activation of passive movement of the patient at t1, t3 (12 weeks), and t4; **(E)** activation of passive movement of healthy control. Color bar = *t*-value. The left side indicates the left hemisphere. L, left; R, right.

Deterministic fiber tracking was conducted after preprocessing using a fiber assignment based on a continuous tracking algorithm with an angle threshold of 30°. Three regions of interest (ROIs) were placed at the precentral gyrus, the posterior limb of the internal capsule, and the cerebral peduncle to reconstruct the corticospinal tract (CST) of interest. The precentral gyrus was extracted from the AAL90 template (8) and spatially registered to an individual fractional anisotropic (FA) map. The individual axial FA map indicated the posterior limb of the internal capsule and the cerebral peduncle (Figure 2A). Tractography was performed on healthy control (Figure 2B) and a t1 and t4 scan of the patient (Figure 2C).

Individual statistical analyses were performed in a matrix design using parameter estimates (based on a general linear model), and contrasts were defined (passive movement vs. rest in the current study). Statistical parametric maps (SPM) of the t statistic were generated and stored as separate images for each subject. The results were analyzed at *p* <0.05 and corrected for multiple comparisons (voxelwise FWE corrected) across the whole brain. In this study, we pre-defined the ROIs by the AAL template (8), including bilateral precentral gyrus, postcentral gyrus, supplementary motor area (SMA), cerebellum (Cb), and parietal lobe (PL) (combining superior and inferior lobe), and premotor cortex (PMC) (combining dorsal premotor cortex and ventral premotor cortex) from the high-resolution sensorimotor area tract template (HMAT) (9). The number of significant active voxels for each pre-defined ROI during passive movement of the left and right hand was obtained. The lateralization index (LI) of the primary sensorimotor cortex (SMC), which includes the precentral gyrus and postcentral gyrus, was used to determine the interhemispheric balance.

#### Rehabilitative treatments

The patient was subjected to a conventional rehabilitation program comprising 60-min physiotherapy (PT) sessions, 45-min occupational therapy (OT), 20-min acupuncture treatment, 20-min neuromuscular electrical stimulation, and 20-min pneumatic gloves for 5 days a week between August 5 and August 26 (t0 to t2). A targeted rTMS protocol was designed for the patient according to the results of the first fMRI and DTI conducted on August 10 (t1). The patient received ten sessions of 1 hz rTMS intervention applied to the contralesional right primary motor cortex (M1) for 10 days between August 13 and August 26. A total of 1,000 stimuli were administered as ten trains of 100 stimuli, with an intertrain interval of 20 s in each session. The stimulation intensity was 100% of RMT.

### Outcomes of rehabilitation

Comprehensive rehabilitation treatments induced a gradual increase in right upper limb motor function and caused a satisfactory functional recovery in the present case. The patient's strength in all upper limb muscles was fully restored (grade 5/5) at discharge on August 26, about 3 weeks after admission to the rehabilitation center. In addition, she could write with her right hand, but her left eye vision had not improved.

#### Clinical scores

Clinical assessment results are presented in Table 1. The BS of the patient increased from 5-4-6 at admission to 6-6-6 at discharge. Moreover, her BI increased from 90 to 100 points. The hand function of the patient improved significantly, as indicated by the ARAT score, which increased from 27 to 54 points. Furthermore, the UPFM score increased from 43 to 61 points, primarily in the distal components of UPFM (wrist and hand), which increased from 10 to 23 points.

**Table 1:** Scores on clinical scales and neurophysiological evaluation results.

**Clinical assessments**	**t0**	**t1**	**t2**	**t3**	**t4**	**Neurophysiological analysis**	**t0**	**t3**	**t4**
UPFM	43	48	61	66	66	Ipsilesional MEP (μV)	/	562	652
UPFM (W/H)	10	12	23	24	24	Ipsilesional RMT (%)	/	53	50
ARAT	19	27	54	57	57	Ipsilesional CMCT (ms)	/	9.35	9.04
BS	5-4-6	5-5-6	6-6-6	6-6-6	6-6-6	Contralesional MEP (μV)	1194	1118	1572
BI	90	95	100	100	100	Contralesional RMT (%)	52	43	45
						Contralesional CMCT (ms)	8.32	8.27	8.14
						RMT ratio	/	1.233	1.111

UPFM (max 66), upper limb Fugl-Meyer Assessment; UPFM (W/H) (max 24), Wrist/Hand component of UPFM; ARAT (max 57), Action Research Arm Test; BS (max 6), Brunnstrom Stages; BI (max 100), Barthel Index; MEP, Motor Evoked Potential (in μV); RMT, Resting Motor threshold (in %, max 100); CMCT, central motor conduction time (in ms); RMT ratio = Ipsilesional RMT/Contralesional RMT.

#### Neurophysiological measures

The ipsilesional MEPs of the patient's FDI were absent at t0 on admission. However, it was induced at the follow-up t3 and t4. The average amplitude of the ipsilesional MEPs increased by 90 μV at t4 compared with that at t3, and the average ipsilesional CMCT at t4 decreased by 0.31 ms. RMT-asymmetry, which was calculated as the ratio of ipsilesional RMT and contralesional RMT, decreased from 1.233 at t3 to 1.111 at t4. The average amplitude of the ipsilesional MEPs at t3 and t4 was significantly lower than the contralesional ones. However, the ipsilesional RMT was higher than the contralesional RMT at t3 and t4. Furthermore, the average CMCT of the affected hemisphere was significantly longer than that of the contralesional hemisphere at t3 and t4. Detailed information is provided in Table 1.

#### Task-based fMRI and DTI measures

CST originating from the precentral gyrus and reaching the cerebral peduncle through the posterior limb of the internal capsule showed no significant difference between the ipsilesional side, the contralesional side of the patient, and the bilateral sides of the healthy control. The mean FA of CST of the healthy control and patient obtained 5 weeks (t1) and 45 weeks (t4) after presenting with stroke are presented in Supplementary Table 1.

The brain activation pattern of passive movement of unaffected hands of the patient was similar to that of healthy control, showing dominant contralateral activation and less ipsilateral activation (Figures 2D,E). However, compared with the passive movement of the unaffected hand of the patient and bilateral hand movement of the healthy control, that of the patient's affected hand consistently displayed more bilateral and wider activation in the primary and secondary sensorimotor cortices. The numbers of significantly active voxels in each ROI during passive movement of the patient and the healthy control are shown in Table 2. The LI of the affected hand movement was 0.19 at t1. The total voxel number of activations of bilateral SMC decreased slightly, and the LI of the affected hand movement increased to 0.35 at t3. Bilateral activations of the affected hand movement were continuously reduced, and the LI of the affected hand movement showed a significant increase (LI = 0.61) at t4. The LI value of the unaffected hand movement at t1, t3, and t4 was 0.99, 0.91, and 0.92, respectively (Table 2), which showed slightly higher than healthy control (LI = 0.88 and 0.84 for the left and right-hand movement, respectively). Notably, bilateral PMC, SMA, PL, and Cb showed hyperactivation at t1 during affected hand movement, and the activation decreased with motor recovery, especially the over-activation of the ipsilateral areas of the secondary motor cortex and non-motor cortex, which almost returned to normal at t4.

**Table 2:** Number of significantly active voxels in each neural region during passive movement for patient and healthy control.

**ROI**	**Left (unaffected) hand movement**				**Right (affected) hand movement**			
	**Patient**			**HC**	**Patient**			**HC**
	**t1**	**t3**	**t4**	**/**	**t1**	**t3**	**t4**	**/**
C_PreCG	478	484	491	400	341	292	196	191
C_PostCG	636	655	647	407	750	550	552	649
C_SMC	1,114	1,139	1,138	807	1,091	842	748	840
C_PMC	221	288	313	180	243	90	92	43
C_SMA	178	179	154	117	318	167	123	111
C_PL	50	60	186	38	301	88	199	198
C_Cb	0	141	70	17	523	305	59	58
I_PreCG	0	0	0	5	136	52	1	0
I_PostCG	4	129	49	48	608	343	157	51
I_SMC	4	129	49	53	744	395	158	51
I_PMC	0	3	1	16	285	81	2	1
I_SMA	46	119	157	120	232	56	7	26
I_PL	0	31	54	34	173	10	0	5
I_Cb	494	597	415	230	956	664	464	282
LI-SMC	0.99	0.80	0.92	0.88	0.19	0.36	0.65	0.89

The LI is calculated for the sensorimotor cortex, defined as the combination of the precentral and postcentral gyrus.

C, contralateral hemisphere to the passive hand movement; I, ipsilateral hemisphere to the passive hand movement; PreCG, precentral gyrus; PostCG, postcentral gyrus; SMC, primary sensorimotor cortex; PMC, premotor cortex; SMA, supplementary motor area; PL, parietal lobe; Cb, cerebellum; LI-SMC, laterality index of SMC.

## Discussion

The present case was a 34-year-old female patient who presented with left eye blindness and a stroke after receiving an HA injection. The lesion was mainly limited to the left precentral gyrus, and the patient showed right arm monoparesis. She underwent 3 weeks of conventional rehabilitation treatment and ten sessions of rTMS intervention in our rehabilitation center 1 month after the stroke onset. The results from clinical assessments showed that the motor function of the patient's right upper limbs was almost completely restored. Neurophysiological analysis showed partial recovery in the ipsilesional MEPs. Task-based fMRI results showed the activation pattern of the affected hand movement was almost restored to normal. The DTI examination showed that the ipsilesional CST of the patient was intact.

### Changes in task-based fMRI and DTI results

Structural and functional MRI results showed that the recovery mechanism of the patient with a lesion mainly restricted to the precentral gyrus would be associated with a complete CST and the recovery of the task-state activation pattern. The activation pattern of the affected hand movement changed from a bilateral pattern to a contralateral one, thus restoring the normal condition. Specifically, this patient's recovery pattern focused on activation in the contralateral SMC with a continued increase in the LI of SMC, accompanied by a decreasing number of over-activated voxels in both hemispheres. This recovery pattern has been reported in the previous study, and another pattern of recovery found in more patients is the continued increase in contralateral SMC activation in subcortical stroke (10). The recovery would be optimal when M1 is not only preserved structurally, as after subcortical as opposed to cortical stroke, but is also capable of enhanced workload (11). However, patients in this study with pure precentral gyrus lesions obtained good recovery through the pattern of progressive focusing. Consistent with previous studies, widespread bilateral recruitment of the secondary motor areas and non-motor cortex occurs first, such as PMC, SMA, and PL, which also happens after cortical stroke (12). Accordingly, the amount of overactivation of these areas declined to normal as recovery took place. In normal subjects, these areas are also involved in hand movement. Thus, bilateral over activation of non-SMC may reflect excess recruitment of a preexisting large-scale distributed motor network rather than genuine reorganization (11). Considering that we applied 1 Hz rTMS to the contralesional M1 to help suppress the over activation of the contralesional hemisphere in this study. As expected, the clinically significant improvement in the patient's hand function was observed after 3 weeks of rehabilitation and ten sessions of targeted rTMS intervention. Notably, although the lesion was mainly located in the precentral gyrus, it was not involved in the original area of the CST pathway in the precentral gyrus, which may also account for the patient's complete CST. Previous studies reported that higher retention of CST leads to better recovery of upper limb function (13–16), and strokes characterized with cortical lesions often have better motor recovery outcomes compared with those with lesions at other sites (17, 18). Structural damage of CST originating from M1 is highly correlated with motor impairments (19), and patients with severe structural pathway damage have lower chances of recovery of upper limb function (20). Therefore, this study's findings may indicate that intact CST would be an essential precondition for good motor recovery. We also suspect that the pattern of recovery and the potential for motor recovery might vary depending on the specific location of the lesion in the precentral gyrus. However, these aforementioned hypotheses are not conclusive enough to be generalized before recruiting more patients with motor cortex lesions and conducting extensive research in the future.

### Role of rehabilitation and rTMS intervention

Rehabilitative training plays an essential role in remodeling modified representation hand function within the perilesional area (21–23). Notably, rTMS may enhance this adaptive plasticity process (24). We observed a progressive recovery of the patient's hand function during 3 weeks of conventional rehabilitation therapy combined with ten sessions of rTMS intervention. In particular, although the ipsilesional MEP of the patient was not initially observed at t0, it was detected at t3 and was observed with a higher average amplitude at t4. Additionally, the ipsilesional CMCT and the RMT-asymmetry of the patient at t4 were lower than those at t3. These results indicated a partial recovery of the ipsilesional central motor conduction velocity and the rebalancing of the interhemispheric excitability. The assumption is that the rehabilitation treatment and 1 Hz rTMS over the contralesional M1 might help the patient regain her motor function through the remodeling of ipsilesional M1 and the restoration of the interhemispheric balance. However, this causality could not be confirmed in this case report. Indeed, in a recent literature review of filler-induced cerebral embolism, it was reported that nearly half of the patients recovered (4.65%) or exhibited improved neurologic manifestations (44.19%), while rehabilitative training as well as the additional rTMS intervention were not involved or mentioned in most cases (25). Moreover, very few studies have explored the application of neuromodulation interventions on pure motor cortical stroke patients. Due to the limitations of our magnetic equipment, we did not test the interhemispheric inhibition (IHI), which is largely mediated by the transcallosal pathways (26), to further explore the interhemispheric asymmetry and inter-cortical inhibition after the rTMS intervention. It would also be interesting to investigate the effects of other neuromodulation protocols, such as cortico-cortical paired associative (ccPAS), which may regulate synaptic strength and induce spike-timing-dependent plasticity in sensorimotor circuits (27–29), on the recovery of motor function after cortical stroke and how these interventions modify the process of cortical plasticity.

### Limitations

This study did not include a control patient who had not undergone rehabilitation. Therefore, the possibility of natural recovery in the progress of the patient's motor recovery could not be ruled out. In addition, the time of the initial neurophysiological evaluation was 5 days before the first fMRI and DTI examinations, and DTI was not conducted at t3.

## Conclusion

In this case report, the patient who presented with motor cortical stroke after a HA filler injection and suffered from hemiplegia obtained an almost complete restoration of her motor function. However, further research is needed to investigate the real benefits and the underlying mechanisms of rehabilitation and neuromodulation approaches to cortical motor stroke.

## Data availability statement

The original contributions presented in the study are included in the article/Supplementary material, further inquiries can be directed to the corresponding author/s.

## Ethics statement

The studies involving human participants were reviewed and approved by Huashan Hospital Affiliated to Fudan University Institutional Review Board (HIRB). The patients/participants provided their written informed consent to participate in this study.

## Author contributions

XT and QY wrote the first draft. XT collected data, performed the literature search, and prepared Figure 1 and Table 1. QY performed data analysis and interpretation and prepared Figure 2 and Table 2. YP and MG prepared Supplementary material. FL, JZ, and KY provided scientific input and clinical support. MF conducted data interpretation and supervised the study. LS conceived the study, was involved in data interpretation, and revised the final manuscript. All authors contributed to the article and approved the submitted version.

## Funding

This work was supported by the National Key R&D Program of China (no. 2020YFC2004200), the National Natural Science Foundation of China (nos. 81974356, 81401859, and 81471651), and the Scientific Research Program of Huashan Hospital (no. 2021QD031).

## Conflict of interest

The authors declare that the research was conducted in the absence of any commercial or financial relationships that could be construed as a potential conflict of interest.

## Publisher's note

All claims expressed in this article are solely those of the authors and do not necessarily represent those of their affiliated organizations, or those of the publisher, the editors and the reviewers. Any product that may be evaluated in this article, or claim that may be made by its manufacturer, is not guaranteed or endorsed by the publisher.
