# Supplementary material for: Multi-modal fMRI and TMS follow-up study of motor cortical stroke caused by hyaluronic acid filler: A case report
Source: Front Neurol. 2022 Sep 8;13:903648. doi: 10.3389/fneur.2022.903648 (PMC9492836; doi:10.3389/fneur.2022.903648)
Supplement: Supplementary file 1 [file Table_1.DOCX]

**Supplementary Material**

**Patient Information**

*Age*: 34 (at the time of Enrolment)

*Sex*: Female

*Handedness*: Right

*Occupation*: Clerk

**Patient Information at the Onset of Stroke (July 2020)**

The 34-year-old Asian woman had no significant medical history. She reported to have received an injection of 1 mL hyaluronic acid (HA) filler into her glabella and forehead using a hollow bore needle at a local private beauty salon, on July 2, 2020 which was administered by a nurse without involvement of a physician. Immediately after HA injection to the left glabella, she developed periocular pain and blurred vision in the left eye and shortly thereafter complete loss of vision in the left eye. She was immediately injected with hyaluronidase into the left glabella and forehead. However, she developed nausea, vomiting, headache, and loss of consciousness within 10 minutes.

She was taken to a local hospital 1h post-injection and admitted to the intensive care unit (ICU). Physical examination at admission showed that the patient was drowsy, with dilated and nonreactive pupil, and lacked light reflex in the left eye. No light perception was observed in her left eye, but the right eye was normal. Her face was swollen and the skin over the forehead and bilateral periorbital regions exhibited petechial hemorrhages and purple discoloration. The initial Glasgow Coma Scale (GCS) score was 13, with negative pathological signs, and no obvious abnormalities were seen on head computed tomography (CT) images. She received dexamethasone 20mg administered intravenously by an ophthalmologist to eliminate edema. In addition, tobramycin dexamethasone eye drops, gatifloxacin eye drops, and pranoprofen eye drops were applied to her left eye. 1h later she developed muscle weakness in her right limbs. Diffusion-weighted imaging (DWI) performed 3h after HA injection revealed acute embolic infarction involving the left frontal and parietal lobes. However, her magnetic resonance angiography (MRA) findings were normal. With a tentative diagnosis of cerebral infarction of undetermined etiology, intravenous mannitol and glucocorticoid were administered to relieve cerebral edema, and oral antiplatelet therapy was also given. She reported headache at the midnight of July 11. Her neurological function was normal, and hence the physician did not give any special treatment.

Her general health condition was stable and thus she was moved to a local rehabilitation center on July 17 because of right hemiplegia. Routine head CT scan was performed on July 20, which revealed hemorrhage in left frontal parietal lobe. However, she did not develop new symptoms of neurological deficit and report any complaints of discomfort. The physician suspended the oral antiplatelet therapy. She received physiotherapy, occupational therapy, and acupuncture for 2 weeks. After these interventions she restored her right lower limb muscle strength, and was able to walk independently without abnormal gait. However, the motor function of her right upper limb was not restored to normal level. The stability and flexibility of her right upper limb and hand was poor, making her unable to hold things with her right hand. Consequently, she was admitted in our rehabilitation center on August 5, 2020 for further management.

**Details at the Time of Enrolment (August 2020)**

At admission the patient was alert and conscious. All vital signs were normal. Neurological examination revealed that her sensations were normal, and her cranial nerves were intact without speech disorders or swallowing problems. Her Mini-Mental Score Examination (MMSE) was 30, and right limbs Brunnstrom Stages (BS) was 5-4-6. Her left forehead skin showed signs of vascular compromise. Ophthalmological examination of the left eye revealed conjunctival hyperemia, dilated pupils, disappearance of light response, pale papillae, surface vascular occlusion, flat omentum and atrophy. Moreover, the left eye nerve fiber layer was thinned and underwent macular atrophy. Further analysis showed that the P100 wave of left visual evoked potential (VEP) was not induced, the amplitude and latency of P100 wave of right VEP were in the normal range. Magnetic resonance imaging (MRI) results showed that the lesion was mainly confined to left precentral gyrus (**Supplementary Figure 1**).

**Details of Rehabilitative Treatments (During August 5 to August 26)**

**Physiotherapy**

Physiotherapy included passive stretching, assisted movement, and resistant activity for the right upper limb muscles and joints. Ball squeezing and release, muscle facilitation techniques were also used.

**Occupational therapy**

Occupational therapy mainly focused on the fine movements of hand and the rotation of wrist and elbow joint. Task-oriented training included writing, holding objects, placing the graphics board, picking beans from board to cup, etc.

**Neuromuscular electrical stimulation**

Transcutaneous neuromuscular electrical stimulation was applied to activate right wrist and finger extensors. Both stimulating electrodes were proximally located over the extensor surface of the forearm. The phase duration was set to 150 µs, pulse frequency was set to 35Hz, and 100 µs interphase interval was applied to a biphasic pulse. To set the stimulation intensity, the amplitude was gradually increased until the electrically-induced contraction developed in the wrist and finger extensors.

**Acupuncture treatment**

An experienced acupuncturist used the following acupoints: LI4, LI10, LI11, and LI15 on the right upper limb of the patient. Locationally, LI4 was at the midpoint of the radial side of the second metacarpal bones between the first and second metacarpal bones on the dorsal of hands. LI10 was located on the dorsal-radial side of the forearms, 2 cun below the cubital crease. LI11 was located at the lateral end of the cubital crease, in the bending depression of the elbow joint. LI15 was at the deltoid of the shoulders, which was located at the depression anterior and inferior to the acromion during arm abduction or extension forward.

**Details of Data Acquisition through TMS**

Before transcranial magnetic stimulation (TMS) examination, the patient was required to relax at least for 2 min. She was asked to sit on the armchair comfortably in a quiet room. TMS was applied using the Yiruide CCY-II TMS instrument (Wuhan, China) with a round coil. The coil was positioned tangentially to the scalp, with the handle pointing posteriorly. The motor evoked potentials (MEPs) were recorded using a self-contained MEP recording system in the Yiruide transcranial magnetic stimulator and were analyzed with an affiliated MEP-analysis software (Wuhan, China). The amplified (100 μV-1mV/div) and bandpass-filtered (10-500 Hz) signals were digitized at a 5000 Hz sampling rate. Surface electromyography (EMG) was recorded by attaching a pair of Ag-Ag/Cl electrodes to the first dorsal interosseous (FDI) muscle of the patient’s hand to assess MEPs. The active electrode of FDI was placed on the belly of the muscle, and the reference electrodes were 2 cm distal from the active electrode. The ground electrode was placed on the wrist. Before stimulation, “hot spots” (where the optimal MEP is elicited by a suprathreshold single-pulse TMS) for FDI in the ipsilesional and contralesional hemisphere were determined and marked on the scalp with a marker to ensure consistent coil placement. Subsequent TMS measurements and rTMS intervention used the same “hot spots”. RMT was defined as the minimal stimulation intensity that can induce at least 5 of 10 consecutive trials in the FDI muscle with MEPs peak-to-peak wave amplitude > 50 μV when the muscle was in relaxed state. The patient’s muscle relaxation was evaluated by visual and EMG monitoring. Ten consecutive MEPs over M1 “hot spots” of FDI were evoked and recorded at an interval > 5 s in both hemispheres as routinely done. The onset and offset of the MEP were defined as the initial deflection of the MEP from baseline and the final regression to baseline. MEP amplitude was measured as the voltage difference from the maximal negative to maximal positive deflection of the MEP (referred to as peak-to-peak amplitude), and MEP latency which reflects the cortical motor conduction time was measured as the period (ms) between stimulus onset and the onset of the MEP. Central motor conduction time (CMCT) for FDI was also recorded. Ten consecutive magnetic stimuli were applied over the lower cervical nerve root to measure the average peripheral motor conduction time. The coil was placed flat on the skin and 2 cm lateral to the C7/C8 cervical spine. CMCT was calculated by subtracting the peripheral motor conduction time from the cortical motor conduction time (1, 2).

**Details of Data Acquisition through Task-based fMRI and DTI**

High-resolution T1-weighted anatomical images, fMRI BOLD images for affected and unaffected passive finger flexion–extension task and diffusion tensor imaging (DTI) data were acquired using Siemens Prisma fit 3.0 Tesla MRI scanner (Siemens, Erlangen, Germany) at Shanghai Key Laboratory of Magnetic Resonance, East China Normal University (Shanghai, China). Additionally, one sex and age matched right-handed healthy subject who reported no history of neurological or psychiatric disorders was also scanned all of these images.

**Data Acquisition**

High-resolution 3D T1-weighted anatomical images were acquired using an MPRAGE sequence with 192 sagittal slices, slice thickness = 1 mm, gap = 0.5 mm, repetition time = 2,530 ms, echo time = 2.34 ms, flip angle = 7°, field of view = 240 mm × 240 mm, and acquisition matrix = 256 × 256. Task-based fMRI data were obtained using an echo-planar imaging sequence with 30 axial slices, thickness=5 mm, no gap, field of view= 220×220 mm, acquisition matrix=64×64, repetition time=3,000 ms, echo time=30 ms, and flip angle=90 °. The DTI data were obtained using a spin-echo single-shot EPI sequence. Diffusion-sensitized gradients were applied along 64 noncollinear directions with a b-value of 1,000 s/mm2. In addition, ten b = 0 image was obtained. The sequence parameters were 70 slices in total, slice thickness = 2 mm, no gap, repetition time = 8,500 ms, echo time = 63 ms, field of view = 224 mm × 224 mm, acquisition matrix = 112 × 112, and flip angle = 90°.

**fMRI task paradigm**

Patients were examined during two fMRI sessions while performing a passive finger flexion–extension task that involved an experimenter clenching the affected hand into a fist; the fMRI sessions occurred before and after the 4-week intervention. A block design was adopted based on IFIS-SA fMRI system (Invivo Corporation, http://www.invivocorp.com/fmri/ifis.php). Following a 30-s rest period, passive movement of affected hand was performed for 30 s. In total, five blocks of passive movement and five blocks of rest period were performed during a single scanning session. The passive movements were performed at 1 Hz following a voice prompt that was controlled by the E-prime program. The amplitudes and frequencies of the passive movements were controlled across subjects or two fMRI sessions because the same examiner in the scanner room performed all passive clenching. An air-filled rubber bulb was placed in the patient’s hand whenever he clutched his hand passively or rested. This rubber bulb was connected to a pressure sensor that was used to visually monitor the force that the examiner applied and maintain consistency of the passive movements during two fMRI sessions. Subjects were instructed to close their eyes throughout. Twenty healthy controls were only scanned for one session while performing the same task for left hands and right hands. Head padding specifically designed for this scanner was used to restrict head motion. All subjects were introduced to the task outside the MRI room and allowed to practice until they were comfortable with the protocol. No mirror movements (associated contralateral hand movements) or synkinesia (associated ipsilateral shoulder or elbow movements) were observed in any patient.

**Preprocessing of DTI Data**

The DTI data were preprocessed using FMRIB’s free software FSL (Oxford Center for Functional MRI of the Brain, Oxford, United Kingdom). All diffusion-weighted images were visually inspected before further processing for apparent artifacts such as subject motion and instrument malfunction. Subsequently, the diffusion-weighted images were registered to the corresponding b = 0 images with an affine transformation to correct for eddy-current distortion and motion displacement. Then, the skulls were removed using the brain extract toolbox. The diffusion tensor was reconstructed using the linear least-square fitting algorithm, which was used for calculating diffusion indices, including the fractional anisotropy (FA), 3 eigenvectors, and the mean diffusivity.

**Preprocessing of Task-based fMRI Data**

The fMRI data was analyzed using the Statistical Parametric Mapping (SPM12, http://www.fil.ion.ucl.ac.uk/spm) and Data Processing & Analysis for Brain Imaging (DPABI, <http://www.rfmri.org/>) (3). Preprocessing of fMRI data was carried out as follows: (i) slice timing was performed to correct the delay in slice acquisition; (ii) realignment was performed to correct rigid-body head movement; (iii) spatial normalization was performed to transform the motion corrected images from individual space to the Montreal Neurological Institute space and resample the images to 3 mm isotropic voxels; and (iv) smoothing was performed to smoothen the normalized images using an isotropic Gaussian filter with a full-width half-maximum of 6 mm.

**Calculation of the Lateralization Index**

The number of voxels of activation in precentral and postcentral gyrus for the contralateral and ipsilateral hemisphere were calculated separately when performing passive hand movement. Furthermore, we calculated the lateralization index between contralateral and ipsilateral precentral and postcentral gyrus activation as follows:

$$LI=\frac{V_{contralateral}-V_{ipsilateral}}{V_{contralateral}+V_{ipsilateral}}$$

Where V means number of activation voxels of precentral gyrus and postcentral gyrus. The LI ranges from -1.0 (ipsilateral activation only) to 1.0 (contralateral activation only), and values close to 0 denoted symmetrical activation. When the passive finger flexion–extension task was performed in healthy controls, more activated contralateral regions were obtained. Therefore, a larger LI value (close to 1) indicated the normal state.

**REFERENCES**

1. Groppa S, Oliviero A, Eisen A, Quartarone A, Cohen LG, Mall V, et al. A practical guide to diagnostic transcranial magnetic stimulation: Report of an IFCN committee. *Clin Neurophysiol*. (2012) 123: 858-82. doi: 10.1016/j.clinph.2012.01.010

2. Rossini PM, Burke D, Chen R, Cohen LG, Daskalakis Z, Di Iorio R, et al. Non-invasive electrical and magnetic stimulation of the brain, spinal cord, roots and peripheral nerves: Basic principles and procedures for routine clinical and research application. An updated report from an I.F.C.N. Committee. *Clin Neurophysiol*. (2015) 126: 1071-107. doi: 10.1016/j.clinph.2015.02.001

3. Yan C, Wang X, Zuo X, Zang Y. DPABI: Data processing & analysis for (Resting-State) brain imaging. *Neuroinformatics*. (2016) 14: 339-51. doi: 10.1007/s12021-016-9299-4

**Supplemental Figure 1.**  Lesion map of the patient


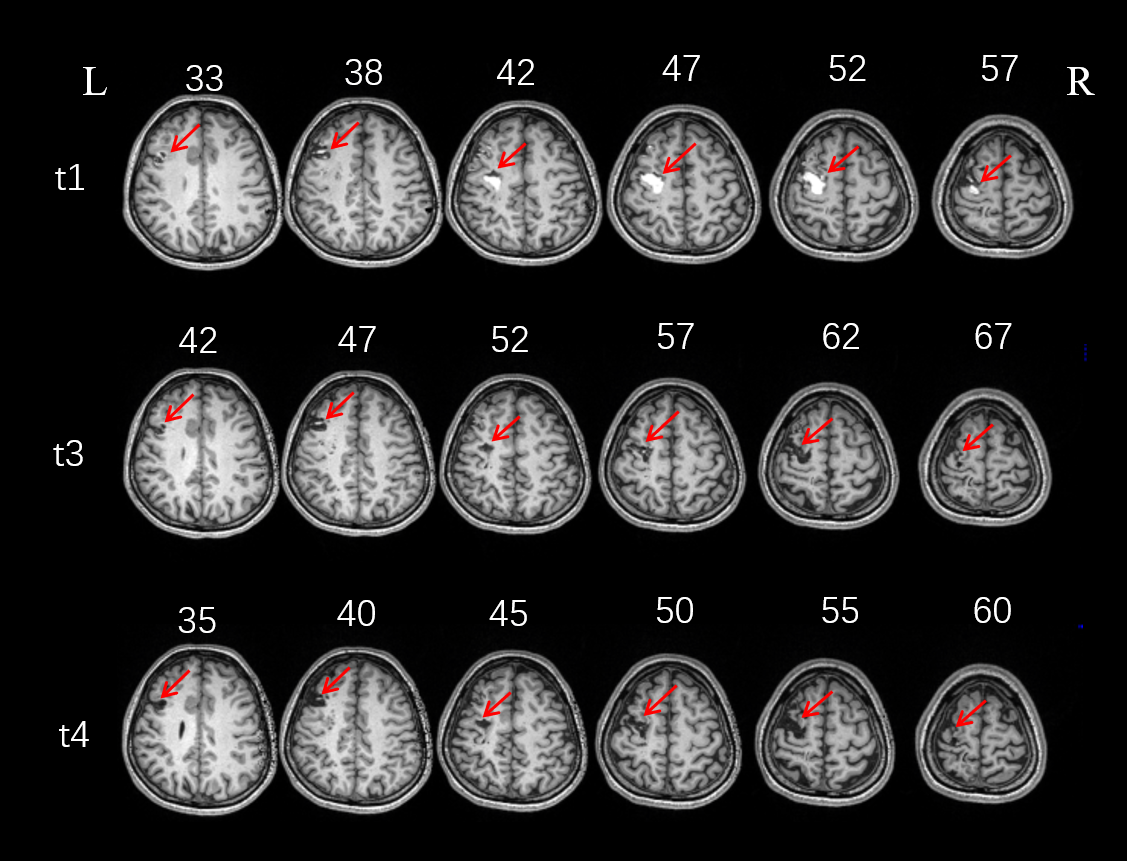


Stroke lesion located mainly in the left precentral gyrus was observed in the axial high-resolution 3D T1-weighted anatomical images. Each column represents an image at a different point in time after stroke (t1 = 5 week, t3 = 12 week, t4 = 45 week). The red arrows point to the location of the lesion.

**Supplemental Table 1.** Mean FA value of CST originating from precentral gyrus in the patient at t1 and t4, and healthy control.

| **Name of fibers** | **Subject** | **Mean FA** |
| --- | --- | --- |
| **Left CST** | Patient_t1 | 0.551±0.145 |
|  | Patient_t4 | 0.563±0.139 |
|  | HC | 0.542±0.138 |
| **Right CST** | Patient_t1 | 0.548±0.141 |
|  | Patient_t4 | 0.569±0.143 |
|  | HC | 0.548±0.139 |

Abbreviations: CST, corticospinal tract; FA, fractional anisotropy.
